# Supplementary figures and images for: GFADE: generalized feature adaptation and discrimination enhancement for deepfake detection
Source: PeerJ Comput Sci. 2025 May 8;11:e2879. doi: 10.7717/peerj-cs.2879 (PMC12192638; doi:10.7717/peerj-cs.2879)

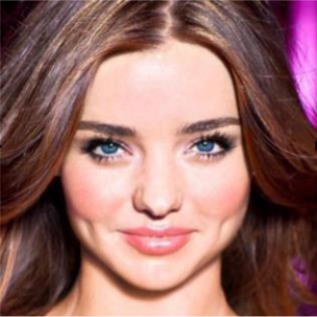

Supplement: Supplemental Information 1 [file peerj-cs-11-2879-s001.zip › Code_GFADE/src/utils/library/000_0000.png]

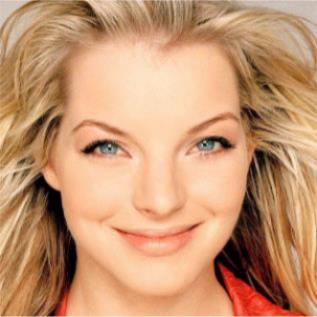

Supplement: Supplemental Information 1 [file peerj-cs-11-2879-s001.zip › Code_GFADE/src/utils/library/001_0000.png]

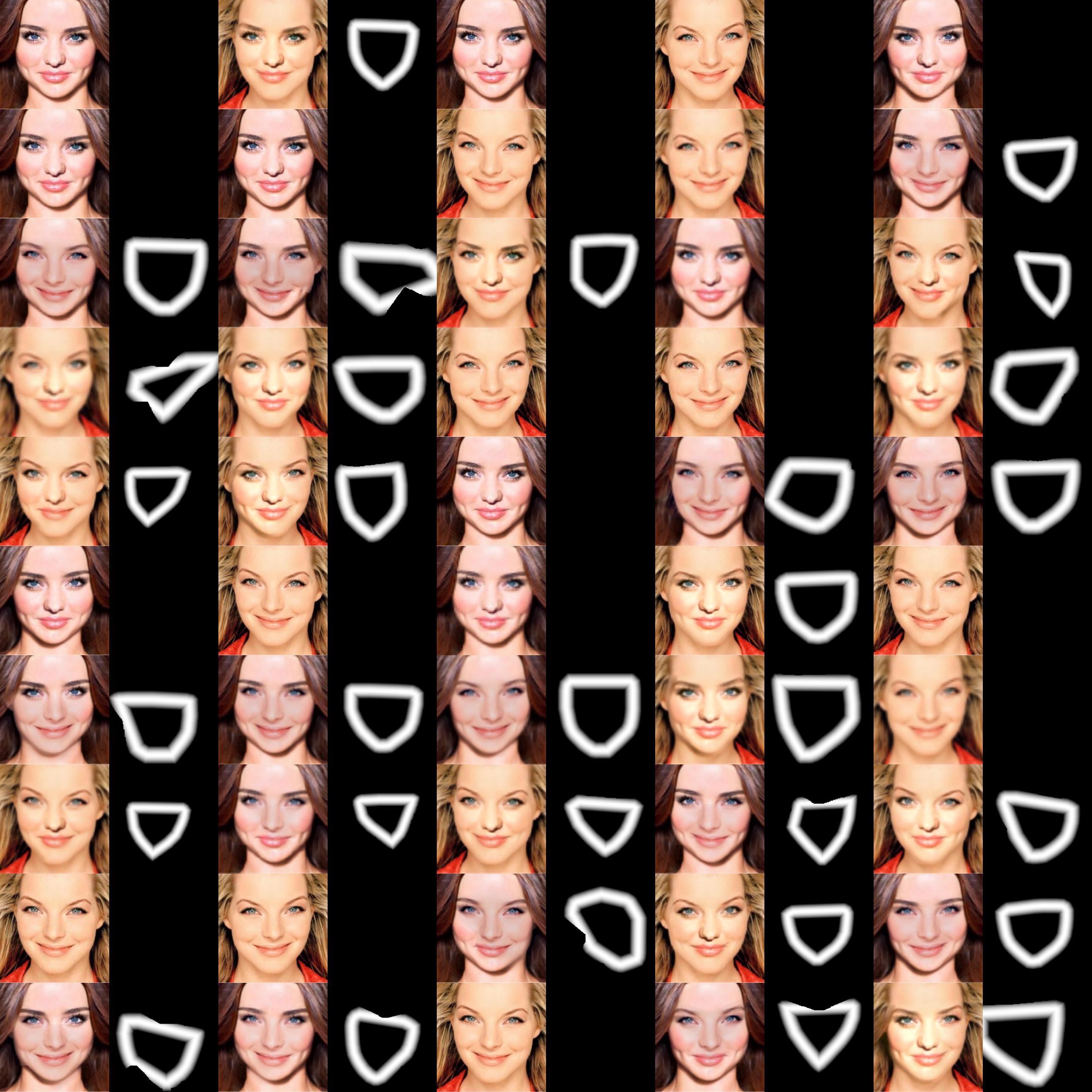

Supplement: Supplemental Information 1 [file peerj-cs-11-2879-s001.zip › Code_GFADE/src/utils/library/all_in_one.jpg]
