# Supplementary material for: GFADE: generalized feature adaptation and discrimination enhancement for deepfake detection
Source: PeerJ Comput Sci. 2025 May 8;11:e2879. doi: 10.7717/peerj-cs.2879 (PMC12192638; doi:10.7717/peerj-cs.2879)
Supplement: Supplemental Information 2 [file peerj-cs-11-2879-s002.docx]

### ****Ethical Statement****

This study aims to explore the potential applications of deepfake technology and investigate defense mechanisms to mitigate misuse risks. The research utilizes only publicly available datasets (e.g., [FaceForensics++]), ensuring compliance with their licensing requirements. No identifiable personal data was included, and no human participants were involved, thus exempting the study from requiring institutional ethical review.

Throughout the study, strict adherence to academic ethical principles was maintained. The generated deepfake content was created solely for research purposes and has been securely stored to prevent any inappropriate dissemination. This research is conducted with the intent to promote the responsible use of technology and strongly opposes the use of deepfake technology for unlawful or unethical purposes.

The authors declare no conflicts of interest related to this study.
